# Creating Connection Beyond Access: A Structured Digital Engagement Platform for Older Adults

**DOI:** 10.1093/geroni/igaf122.4349

**Published:** 2025-12-31

**Authors:** Soohyoung Rain Lee, Andrea Maxi, Sharon Turnage, Cathy Sasscer, Sarah Kaufman, Hang Liu, Ana Semedo, Grace Yi

**Affiliations:** Yeshiva University, New York, New York, United States; Yeshiva University, New York, New York, United States; Yeshiva University, New York, New York, United States; Yeshiva University, New York, New York, United States; Yeshiva University, New York, New York, United States; Selfhelp, New York, New York, United States; Selfhelp, New York, New York, United States; California State University, Fullerton, Fullerton, California, United States

## Abstract

Loneliness and depressive symptoms are persistent concerns in later life, particularly for those with functional or sensory limitations. Digital programs may help, yet evidence on feasibility and mental health correlates in diverse samples is limited. This study evaluated the Virtual Senior Center (VSC), a facilitator-led, routine-based online program, examining loneliness, depressive symptoms, digital confidence, and perceived benefits, supplemented with focus group interviews. Twenty-three racially diverse older adults completed baseline surveys after enrollment and participation in at least one session. Measures included the UCLA Loneliness Scale, Geriatric Depression Scale-10, digital confidence, functional limitations, and participation. Descriptive statistics, Mann-Whitney U, and Spearman’s ρ were used. Focus groups were analyzed using rapid content analysis. Participants reported moderate loneliness (M = 41.4, SD = 15.0) and mild depressive symptoms (M = 2.3, SD = 2.2). Digital confidence was negatively associated with loneliness (ρ = -.49, p = .02) and depressive symptoms (ρ = -.54, p = .01). Functional limitations were associated with higher depressive symptoms (ρ = .53, p = .01), but not loneliness, and did not reduce program use. Focus group themes emphasized the value of structured routines and consistent opportunities for peer connection, making VSC a meaningful part of weekly life. Findings suggest that digital confidence supports emotional well-being and that structured, inclusive programs can sustain engagement even among older adults with impairments. By fostering routine, familiarity, and continuity, areas where many digital interventions failed to provide, VSC demonstrates potential to reduce isolation and support aging well in the community.

